# New Insights in the Sugarcane Transcriptome Responding to Drought Stress as Revealed by Supersage

**DOI:** 10.1100/2012/821062

**Published:** 2012-05-02

**Authors:** Éderson Akio Kido, José Ribamar Costa Ferreira Neto, Roberta Lane de Oliveira Silva, Valesca Pandolfi, Ana Carolina Ribeiro Guimarães, Daniela Truffi Veiga, Sabrina Moutinho Chabregas, Sérgio Crovella, Ana Maria Benko-Iseppon

**Affiliations:** ^1^Department of Genetics, Federal University of Pernambuco (UFPE), 50670-901 Recife, PE, Brazil; ^2^Biotechnology Division, Sugarcane Technology Center (CTC), 13400-970 Piracicaba, SP, Brazil

## Abstract

In the scope of the present work, four SuperSAGE libraries have been generated, using bulked root tissues from four drought-tolerant accessions as compared with four bulked sensitive genotypes, aiming to generate a panel of differentially expressed stress-responsive genes. Both groups were submitted to 24 hours of water deficit stress. The SuperSAGE libraries produced 8,787,315 tags (26 bp) that, after exclusion of singlets, allowed the identification of 205,975 unitags. Most relevant BlastN matches comprised 567,420 tags, regarding 75,404 unitags with 164,860 different ESTs. To optimize the annotation efficiency, the Gene Ontology (GO) categorization was carried out for 186,191 ESTs (BlastN against Uniprot-SwissProt), permitting the categorization of 118,208 ESTs (63.5%). In an attempt to elect a group of the best tags to be validated by RTqPCR, the GO categorization of the tag-related ESTs allowed the *in silico* identification of 213 upregulated unitags responding basically to abiotic stresses, from which 145 presented no hits after BlastN analysis, probably concerning new genes still uncovered in previous studies. The present report analyzes the sugarcane transcriptome under drought stress, using a combination of high-throughput transcriptome profiling by SuperSAGE with the Solexa sequencing technology, allowing the identification of potential target genes during the stress response.

## 1. Introduction

Sugarcane (*Saccharum* spp.) is an outstanding crop throughout the tropical regions of the world [1]. It represents an important food and bioenergy source, being cultivated in many tropical and subtropical countries [2], and covering more than 23 million hectares worldwide, with a production of 1.6 billion metric tons of crushable stems [3]. This crop is responsible for almost two thirds of the global sugar production [1]. Brazil, the world's largest sugarcane producer, processed and generated in 2008 about 31 million tons of sugar [4]. In contrast to most plants, sugarcane stores sucrose—rather than polymeric compounds such as starch, proteins, or lipids—as the primary carbon and energy reserve [1]. Hence, sugarcane byproducts have received greater attention, due to their multiple uses, with the ethanol generation being highlighted, as an important renewable biofuel source [5]. Moreover, the bagasse of sugarcane has been largely used for energy cogeneration at distilleries, production of animal feed and also for paper production [6]. Nevertheless, similarly to other meaningful agronomical crops, sugarcane cultivation faces considerable losses due to inappropriate or unfavorable edaphoclimatic conditions.

Abiotic stresses are among the main causes of major crops worldwide productivity losses [7], causing negative impacts on crop adaptation and productivity. In this scenario, drought figures as the most significant stress and is considered an extremely important factor when it comes of losses in the productivity of sugarcane [8]. Several plant biotechnology programs have been initiated aiming to increase drought stress tolerance in crop plants using genetic engineering and traditional breeding [9]. Although breeding activities have provided significant progress for the understanding of the physiological and molecular responses of plants to water deficit, there is still a large gap between yields in optimal and stress conditions [10]. For this purpose, case-sensitive methods are demanded, not only to discover new genes associated to those stress conditions, but also to effectively detect differentially expressed genes on a drought tolerant variety. The identification and expression profile of such responsive genes may be helpful to unravel the basic mechanism of stress tolerance [11]. In this sense, previous works uncovered genes associated to important roles in stress perception, signal transduction, and transcriptional regulatory networks in cellular responses, useful for the improvement of stress tolerance in plants by gene transfer [12, 13].

Molecular approaches concerning drought and salinity performance in sugarcane were carried out using techniques based on molecular hybridization such as *Suppression Subtractive Hybridization *(SSH) [11] and micro-/macroarrays [14]. In general, the main limitations of these methods are their low sensibility and specificity [15]. Among the methodologies for transcriptomic analysis, the SuperSAGE [16] approach represents one of the most recent and informative methods [17], especially with its association to the high-performance sequencing platforms [pyrosequencer (454 Roche), Solexa (Illumina), and SOLiD (Applied Biosystems)]. SuperSAGE regards an evolution of the traditional *Serial Analysis of Gene Expression* [18] generating longer (26 bp) tags and thus allowing most reliable annotation analysis. Since, it is an open architecture method (i.e., allowing the discovery of new genes), it presents the potential to provide a global and quantitative gene expression analysis, based on the study of the entire transcriptome produced in a given time and tissue, under a given stimulus. Additionally, SuperSAGE permits a simultaneous analysis of two interacting eukaryotic organisms, full-length cDNAs amplification using tags as primers, potential use of tags via RNA interference (RNAi) in gene function studies, identification of antisense and rare transcripts, and identification of transcripts with alternative splicing [19]. Besides, this method has been recently associated to the next generation sequencing technologies, allowing a less expensive and faster covering of the analyzed transcriptomes, permitting a deep insight of the modulated responses under different physiological conditions. The association of SuperSAGE with the rapid advances in high throughput sequencing opened the possibility of performing genome-wide transcriptome studies in non model organisms. Additionally, this technique has been successfully applied in plant species such as rice [16], banana [20], chickpea [21, 22], chili pepper [23], tobacco [24], and tropical crops (cowpea, soybean, sugarcane; [25]). In the present work, we profit from the high resolution power of SuperSAGE coupled to the Illumina sequencing to characterize the transcriptome of drought-stressed sugarcane roots after 24 hours of submission to this stress, aiming to elect a best group of tags to be validated by RTqPCR.

## 2. Methodology

### 2.1. Identification of Drought-Tolerant and Sensitive Sugarcane Accessions

For the selection of the drought-tolerant and sensitive accessions used in the present evaluation, a previous assay was carried out in order to identify contrasting genotypes for these features. For this purpose, 20 commercial sugarcane varieties (CTC 1 to 15, SP83-2847, SP83-5073, CT94-3116, SP90-1638, and SP90-3414) from CTC (Sugarcane Technology Center, Piracicaba, Brazil) were evaluated. Among these, the four above-mentioned varieties were used as a standard for the interpretation of results, including two varieties (SP83 and SP83-2847-5073) identified as drought-tolerant and other two (SP90 and SP90-1638-3414) indicated as drought-sensitive based on field empirical observations performed by specialized technicians during several years in sugarcane commercial fields.

For this assay, mini-cuttings from the 20 varieties above were planted in 50 L pods containing inert substrate (Plantmax) in order to allow the slow increase of water deficit by removing irrigation. Tests were performed with six-months-old plants under greenhouse conditions and the treatments included plant permanently irrigated (without stress), suppression of irrigation for three days (72 hours stress), suppression of irrigation for 10 days (240 hours stress), and suppression of irrigation for 20 days (480 hours stress). Physiological measurements applied in all treatments included chlorophyll content using an SPAD-507.B Chlorophyll Meter; analysis of chlorophyll fluorescence ratio between variable and maximum chlorophyll-*a* (Fv/Fm); estimation of chlorophyll content with a fluorometer; determining the relative water content. For the parameters of chlorophyll-*a* fluorescence and chlorophyll content, three measurements were taken from three plants from each treatment. Data analysis was performed by comparing the percentage change considering the parameters mentioned above. After this assay, four drought-tolerant and four sensitive accessions could be selected according to the parameters used, revealing a gradient of water stress tolerance among the varieties analyzed. Considering the classification of the standard varieties identified previously as drought-tolerant (SP83-2847 and SP83-5073) and drought-sensitive (SP90-1638 e SP90-3414) and also considering the measurements taken after stress under glasshouse conditions (these results will be presented in a separated manuscript) four varieties were considered as drought-tolerant (CTC15, CTC6, SP83-2847, and SP83-5073) and other four as drought-sensitive (CTC9, CTC13, SP90-3414, and SP90-1638).

### 2.2. Drought Stress Application and the SuperSAGE Libraries

Plants of each selected accession were grown under glasshouse conditions in 40 L pods, in randomized experimental design (comprising six repetitions) under daily irrigation until the age of three months. After that, part of the material was submitted to drought by interruption of irrigation during 24 hours. Roots of both, stressed and nonstressed plants, were collected and frozen in liquid N_2_, being maintained in a deep freezer until total RNA extraction using Trizol (Invitrogen). The extracted samples were quantified by spectrophotometry, digested with DNAse and purified with the aid of the RNeasy Mini kit (Qiagen). The samples were quantified again by spectrophotometry, allowing the composition of the bulks using equimolar amounts of poli-A^+^ messenger RNA, for all treatments. Four libraries have been generated: TD (bulk of four tolerant accessions under stress); TC (bulk of four tolerant genotypes without stress, as tolerant negative control); SD (bulk of four sensitive materials after stress); SC (bulk of nonstressed sensitive accessions, as sensitive negative control). The procedures for SuperSAGE library generation followed Matsumura et al. [26], including the attachment of library-specific adaptors carried out by GenXPro GmbH (Frankfurt am Main, Germany) allowing the identification of library-specific reads after SOLEXA sequencing.

### 2.3. Statistical Analysis and Tag-Gene Annotation

The 26-bp tags were extracted from each library. Singlets (reads appearing only once) were excluded from the present evaluation. Statistical tests were applied to the remaining tags (Audic Test, Claverie; *P* ≤ 0.05) with aid of the DiscoverySpace 4.1 software [27] regarding the four contrasting treatments [T (TD *versus* TC); S (SD *versus* SC); D (TD *versus* SD); C (TC *versus* SC)]. The tests allowed the identification of the total number of expressed unitags (or tag species) for each situation and contrast, as well as the differentially expressed tags, including up- (UR) and downregulated (DR) tags. The tag-gene annotation was performed by independent evaluations via BlastN [28] against different EST databases: NCBI: (i) dbEST including only *Saccharum* ESTs; (ii) Gene Index (including *Arabidopsis thaliana*, AtGI 15.0, and Poaceae species: *S. officinarum*, SOGI 3.0; *Sorghum bicolor*, SBGI 9.0; *Zea mays*, ZMGI 19.0; *Panicum virgatum*, PAVIGI 1.0; *Oryza sativa*, OsGI 18.0; *Triticum aestivum*, TAGI 12.0; *Hordeum vulgare*, HVGI 11.0; *Festuca arendinaceae*, FAGI 3.0; *Secare cereale*, RYEGI 4.0); and (iii) KEGG (including *A. thaliana* and Fabaceae ESTs)]. Valid BlastN alignments were considered when the following parameters were observed: score from 42 to 52; integrity of the CATG sequence at the 5′ end; plus/plus alignments. Inferences about the modulation of a specific tag (Fold Change; FC) were carried out considering the ratio of the observed frequencies of a given library in relation to the other.

### 2.4. Gene Ontology of SuperSAGE Hits

Matching ESTs to the analyzed tags were categorized via GO using the software Blast2GO [29] after BlastX alignment against the Uniprot-SwissProt protein database (e-value ≤ e^−10^). ESTs related to the GO subcategories concerning abiotic stress response (to water deprivation, GO: 0009414; to heat/cold, GO: 0009408/GO: 0009409; to osmotic stress, GO: 0006970, to oxidative stress, GO: 0006979, to abscisic acid stimulus, GO: 0009737; to jasmonic acid stimulus, GO: 0009753) were identified, as well as UR tags related to these classes. Sets of UR tags considering the different contrasting situations (T, S, D, and C) were annotated, generating Venn diagrams, aiming the visualization of specific or shared tags considering the different treatments.

## 3. Results and Discussion

### 3.1. Qualitative and Quantitative Analysis of the SuperSAGE Libraries

The four SuperSAGE libraries produced 8,787,315 tags, from which 1,862,064 (21.2%) regarded singlets (tags sequenced only once), and were excluded from this evaluation. The most representative libraries considering the number of tags were TC (drought-tolerant control; 2,516,454 tags) and SD (drought-sensitive under stress; 2,133,587 tags), while the less representative were TD (drought-tolerant under stress; 750,226 tags) and SC (drought-sensitive control; 762,492 tags). The coverage of the transcriptome by the tags was estimated considering the total number of tags per genotype (3,266,680 for the tolerant bulk and 2,896,079 for the sensitive bulk) in relation to the number of expected transcripts per cell. The total number of average-sized transcripts was estimated to range from 100,000 [30] to 500,000 [31] per cell in higher plants. Considering the high value (500,000), the coverage provided by the tags in relation to the sugarcane transcriptome was 6.5 times higher for the tolerant bulk and 5.8 for the sensitive bulk, that is, the number of expected single copy transcripts per cell should be represented by their tags in the absolute frequencies of around six in each library. Taking the less represented libraries (TD and SC) in account, the coverage of the transcriptome regarded 1.5 times higher for both, tolerant and sensitive bulks. Considering this value, we established the *n* < 2 frequency as cutoff threshold, allowing the exclusion of singlet tags. Coverage of this magnitude allowed a comprehensive evaluation of a given transcriptome, also including rare transcripts expressed during the response to the evaluated stress.

Taking all valid tags (*n* ≥ 2) into account, a total of 205,975 unitags remained for evaluation. In a recent approach, Yamaguchi et al. [32] observed similar amounts (*≈*190,000 unitags) in the roots of *Solanum torvum* under heavy metal stress (CdCl_2_ 0.1 *μ*M). The high number of unitags, here observed, shows the diversity of transcripts (and expressed genes), possibly also reflecting the allopolyploid nature of sugarcane, since tags diverging in a single nucleotide were considered to be distinct unitags. It has been speculated that, in some cases, unitags could be the result of artifacts generated by the amplification process during library construction [33] or incomplete digestion of the synthesized cDNA by the *Nla*III enzyme [34], and also by PCR amplifications associated to innate features of the sequencing technology [32]. In order to minimize error sources, some precautions were taken during library development in this study, including double digestion of the total RNA extracted with DNAse, double digestion with the *Nla*III enzyme, and exclusion of singlet tags. An additional way to minimize potential errors would be the exclusion of unitags related to other similar sister-tags, grouping them to other most frequent, so called mother-tags. On the other hand, this procedure would eliminate transcripts bearing important single nucleotide polymorphisms (SNPs). Still, another possibility would be to establish a minimum frequency (*n*) of a given tag to be considered valid. In the present work, only canonical tags were accepted, with complete adapter sequences (removed by *in silico* procedures) bearing the full CATG restriction site and with *n* > 2. A more stringent value (*n* > 10) was adopted by Yamaguchi et al. [32], to reduce the number of unitags per library (from 300,000 to 450,000) for each 33 thousand tags, in an attempt to reach the number of expected genes for model species as rice (32,000 genes) and *A. thaliana* (26,000 genes). However, this procedure impairs the identification of rare and alternative transcripts that possibly play important roles in the cell metabolism.

Statistical analysis considering *P* ≤ 0.05 (Audic-Claverie test) among libraries permitted the identification of differentially expressed tags including up- (UR) or downregulated (DR) tags for the four contrasting situations (**T**; **S**; **D**; **C**), as shown in Table 1.

### 3.2. Primary Annotation of SuperSAGE Tags

Relevant BlastN alignments comprised 567,420 tags (75,404 unitags with 164,860 different ESTs). Details about the results obtained after alignment to different databases are not itemized here, since this is not the aim of the present evaluation. Despite that the choice of the databases and the adopted criteria allowed the following: (a) the identification of ESTs related to most tags, preferentially concerning sequences from sugarcane or taxonomic the related species sequences; (b) annotation of a considerable number of tags considering a minimum alignment of 21 bp (similar to a LongSAGE tag); (c) identification of tags with perfect alignments (100% identity) or with a maximum of a single mismatch among tag and EST, important for future development of primers; (d) avoidance of plus/minus alignments, minimizing false NATs (natural antisense transcripts).

The strategy of considering the alignments without the election of a best hit allowed the maximization of annotation chances, since no alignment was disregarded in the acceptable score range. Thus, alignments with annotated ESTs could be more informative than similar alignments with a slightly superior score in relation to nonannotated ESTs. Moreover, tags aligned with distinct ESTs could be analyzed, minimizing the chance of a wrong choice that could compromise the validation of the expression results, especially considering that they are used as targets for RTqPCR primer design. In this context, seeking the maximization of the annotation procedures, the use of the Gene Index database for tag identity annotation was carried out trying to circumvent at least two limitations, when compared with the partial dbEST bank additionally used: (a) no need of clusterization concerning ESTs deposited at dbEST, since the Gene Index project provides tentative clusters (TC); (b) best functional annotation, with the Uniref100 (Uniprot) bank as reference. Thus, in view of the posterior need of primer design for RTqPCR and data validation of SuperSAGE tags, alignments with tolerance of a maximum of a single mismatch (TSM) tag-hit represented up to one third (186,191 or 32.8%) of the data, indicating high identity among 26 bp tags and similar ESTs, since a minimum of 21 bp alignment (size of a LongSAGE tag) was considered relevant. Almost all valid alignments (471,672 or 83.12%) regarded *Saccharum* spp. (partial dbEST) and *S. officinarum* (Gene Index SOGI), as expected. TSM alignments restricted to these databases comprised 163,742 tags. Considering TSM alignments with sequences of the SOGI only, from 26,884 ESTs, 73.0% presented informative gene descriptions and/or their functions, allowing the identification of molecular targets and gene-feature association. Despite of the higher number of TSM matches concerning alignments with dbEST sequences (136,858), the EST annotation was not informative for most contemplated ESTs (97.0%). To overcome this deficiency, the Gene Ontology categorization proved to bring a valuable contribution.

### 3.3. Functional Categorization of SuperSAGE Tags

**Figure 1:**
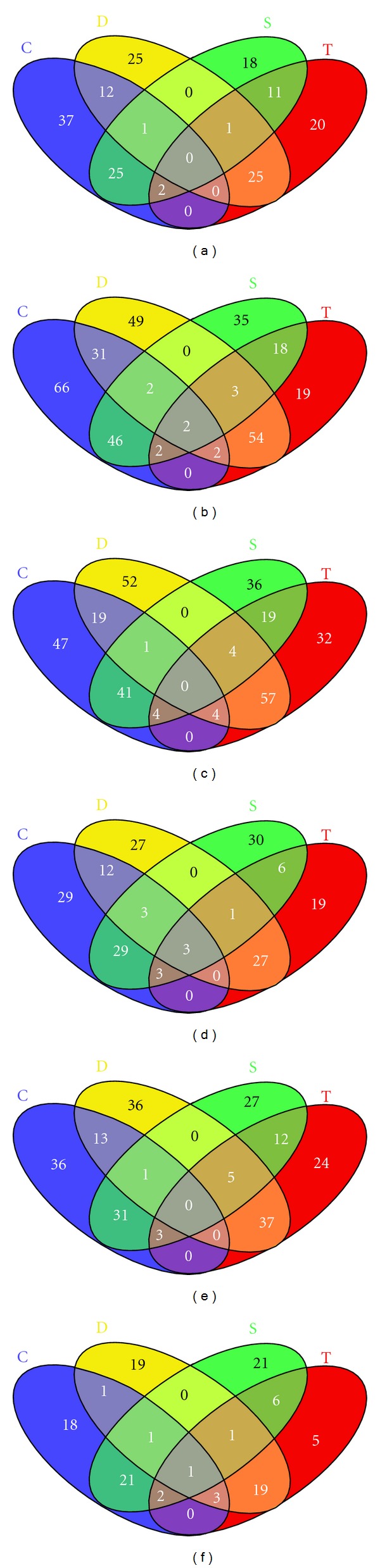
Venn diagrams with numbers of differentially upregulated (UR) tags from sugarcane roots (*P* ≤ 0.05) under drought stress, considering different comparisons between SuperSAGE libraries [contrasts: T (TD *versus* TC); S (SD *versus* SC); D (TD *versus* SD); C (TC *versus* SC)]. UR tags associated with gene ontology (GO) response to (a) water deprivation; (b) heat/cold; (c) osmotic stress; (d) oxidative stress; (e) abscisic acid stimulus; (f) jasmonic acid stimulus. Libraries: TD (drought tolerant bulk under stress); TC (tolerant bulk control); SD (drought sensitive bulk under stress); SC (sensitive bulk control).

**Figure 2:**
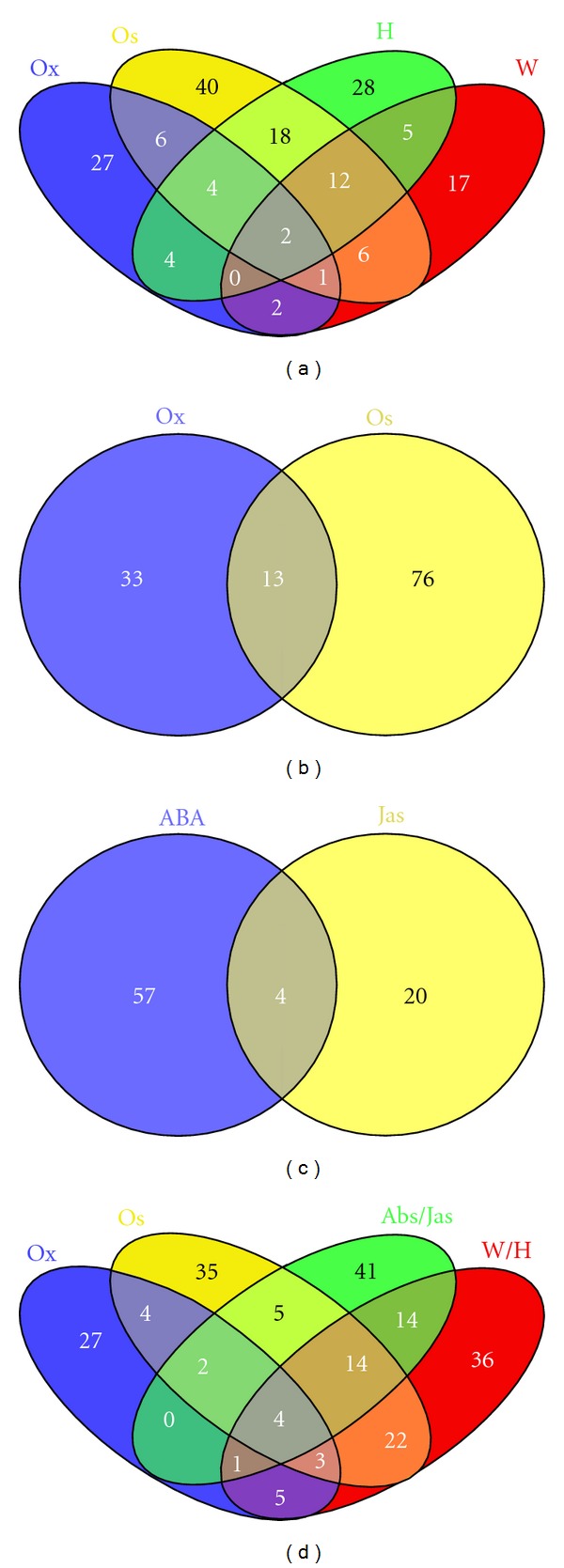
Venn diagrams with numbers of SuperSAGE tags overexpressed (*P* ≤ 0.05) in sugarcane roots under drought stress, considering different tag sets related to gene ontology (GO) subcategories associated in response to: W (water deprivation), H (heat/cold), Os (osmotic stress), Ox (oxidative stress), ABA (abscisic acid stimulus), Jas (jasmonic acid stimulus).

BlastX evaluations (e-value ≤ e^−10^) of the 186,191 ESTs (diverse databases and TSM alignments) against the peptide Uniprot-SwissProt bank allowed the characterization of 118,208 ESTs (63.5%) that presented at least one GO reference. From this categorization, the Biological Process (BP) subcategories in response to abiotic stress were considered more informative to evaluate the sugarcane response to drought conditions. The first interesting indicators were UR tags associated to EST in the BP subcategories responding to water deprivation (GO: 0009414), heat (GO: 0009408), cold (GO: 0009409), osmotic stress (GO: 0006970), oxidative stress (GO: 0006979), abscisic acid stimulus (GO: 0009737), and jasmonic acid stimulus (GO: 0009753). By the analysis of the UR tags observed in the above-mentioned subcategories (Table 2), it was possible to generate the Venn diagrams presented in Figure 1, where Figure 1(a) represents the UR tags evaluated in the contrasting situations T (TD *versus* TC; 20 tags) and D (TD *versus* SD; 25 tags), both important for future gene validation. The first case (T) related to tags from the tolerant bulk induced after water deficit when compared with the bulk control; the second group refers to induced tags from both bulks submitted to drought stress (tolerant *versus* sensitive), with higher expression (UR) in the tolerant bulk. The first group exhibited 17 non annotated tags and only three identified genes (encoding 18S ribosomal RNA, membrane integral protein, and viviparous-14). The second group included 17 tags without annotation and other eight bearing descriptions (18S ribosomal RNA gene (two tags); ABA responsive element binding factor 2; Auxin-induced protein; DRF-like transcription factor DRFL2a; ERF/AP2 domain containing transcription factor; GST; RAPB protein) are discussed latter in this manuscript. Additionally, 11 tags are worth mentioning, since they were UR in both tolerant (T) and sensitive (S) comparisons after stress (Figure 1(a)), when compared to the expression of the respective controls. Despite of being not genotype-dependent, these tags may influence positively in the plant adaptation process under drought stress. Such results and other for similar subcategories are presented in Table 2. This table comprises the total number of UR tags induced in the tolerant bulk under stress, highlighting the exclusive (T comparison) or differentially expressed tags in comparison to the sensitive bulk (D comparison), bringing interesting candidates for validation via RTqPCR. Since the same tags may be involved in different stresses, the identified tags (exclusive in T and common in the comparisons T and D; Table 2) may not be exclusive of a given condition or response. Thus, the total number of UR tags (alone or in combination) in response to water deprivation (W), heat/cold (H), osmotic stress (Os), and oxidative stress (Ox) is presented in Figure 2(a). Likewise, the number of tags induced in response to osmotic and oxidative stress is presented in Figure 2(b), while the tags responsive to hormonal stimuli (abscisic and jasmonic acids) is shown in the Figure 2(c), and a Venn diagram showing all the categories is presented in Figure 2(d).

In relation to the 213 UR tags, including the exclusive ones from the T contrast and those presented in both T and D contrasts (Table 2), the gene-function annotation together with the GO descriptions were available for 68 of them, while 145 tags remained unknown candidates. The annotations of these 68 UR tags and respective GO subcategories, as well as the fold change (FC) for both most relevant contrasts (T and D), are listed in Table 3. Some of them will be further addressed.

#### 3.3.1. Response to Hormone Stimulus

Response to hormonal stimulus, such as jasmonic (JA) and abscisic acid (ABA), together with other plant hormones, as salicylic acid (SA) and ethylene (ET), form a complex network that plays major roles in disease resistance and response to abiotic stresses, including drought [35, 36]. In our study, 21 potential hormone-responsive tags were identified (Table 3) and some of them are thereafter discussed.

**(a) ZIM Motif Family Protein**

According to the database of *Arabidopsis *transcription factors (DATF; http://datf.cbi.pku.edu.cn/index.php), this short motif is associated to a panel of plant transcription factors and JA signaling, which is among the most important defense-related signals in plants, acting under environmental stresses, such as UV radiation, osmotic shock, heat, and drought [37]. Examining a jasmonate-insensitive 3 (jai3-1) mutant gene, Chini et al. [38] identified a novel family of jasmonate-regulated nuclear targets of SCFCOI1, named jasmonate ZIM-domain (JAZ) proteins repressing JA signaling and targeted by the E3-ubiquitin ligase SCFCOI1 for proteasome degradation. The overexpression of this hormone activated a damping mechanism concerning the JA signaling cascades after stress initiation [39]. In our evaluation, five UR tags were hormone related, with one candidate (SD108270) presenting expressive fold change in both contrasts (FC_T_ = 6.4 and FC_D_ = 2.6; Table 3).

**(b) Chromatin-Remodeling Factor**

CHD3 has been implicated in the repression of transcription [40]. Association of these proteins to drought-responsive genes was related during *Arabidopsis* seed germination process by regulating the ABA-dependent and gibberellic acid (GA) dependent responses, modulating the plant reaction to mild osmotic stresses and limiting the expression levels of transcription factors, preventing a maladapted growth arrest. In other words, it refines the pace of seed germination in response to ABA and maintains embryonic characters silent in response to GA [41]. Our results indicate a differential expression of CHD3 also in roots of adult sugarcane plants undergoing water deficit, with two UR tags (SD75453 and SD123546) with FC values of 1.6 (SD123546; FC_T_) and 2.8 (SD75453; FC_T_ = FC_D_; Table 3).

**(c) AP2/EREBP**

It is a large family of plant transcriptional regulators that plays key roles in the development and environmental stress response pathways. Transcription factors encoded by AP2/EREBP genes contain the highly conserved AP2/ERF DNA binding domain [42] constituting a plant supergene family [43] subdivided into five subfamilies according to the number of AP2/ERF motifs [44]. The AP2/EREBP subgroup induced by biotic and abiotic stresses was identified by Sharoni et al. [45]. Among the upregulated genes, 52 were induced in response to diverse abiotic stress, such as cold, drought, and salt. Lin et al. [46] working with a full-length cDNA *OsEBP2* (ethylene-responsive-element binding protein2) in japonica rice leaves infected by blast fungus *Magnaporthe grisea* observed that *OsEBP2* responded transiently to the treatments with methyl jasmonate (MeJA), ABA, and ethophen (ethylene generator). In our analysis, a UR tag was annotated as *APETALA 2/ethylene response element binding protein* (AP2/EREBP) showing expressive modulation (FC_T_ = 2.9 and FC_D_ = 2.3; Table 3). Additionally, one UR tag (SD286424; FC_T_ = 1.4 and FC_D_ = 2.6; Table 3) annotated as *AP2/ERF domain containing transcription factor* was associated to our WH group (response to water deprivation + response to heat/cold; Table 3), indicating an important candidate for validation, since the overexpression of an ERF transcription factor GmERF3 from soybean in tobacco plants raised the tolerance to salinity (up to 400 mM, NaCl) and drought [47] in transgenic plants.

#### 3.3.2. Response to Water Deprivation, Oxidative and Osmotic Stress

In our analysis, 47 potential stress-responsive UR tags with acceptable annotation were identified (Table 3) and some of them deserve special mentioning when considering their GO categorization and the fold change data.

**(a) Glycine-Rich RNA Binding Protein (GRP) Superfamily**

This superfamily, characterized by the presence of a glycine-rich domain arranged in (Gly)n-X repeats, was recently reviewed by Mangeon et al. [48] that highlighted the diversity in structure, expression pattern, and subcellular localization, suggesting that these proteins perform different functions in plants, such as processing, transport, localization, stability, and translation of mRNA molecules. This supposition is consistent with literature data regarding GRPs and biotic and abiotic stresses [49, 50]. Wang et al. [50] analyzing the transcriptome of *Malus prunifolia* (an apple relative with strong drought tolerance) identified a GRP (*MpGR-RBP1*) expressed in roots and leaves, which plays a role in the response to plant dehydration. Among the most representative tags found to be water-deprivation responsive in our analysis, nine tags with FC ranging near 1.1 up to 25.0 (both FC_T_ and FC_D_; Table 3) in roots showed to be upregulated in the drought-tolerant bulk under stress when compared to nonstressed control (TD *versus* TC) or in relation to the drought-sensitive bulk also under stress (TD *versus* SD).

**(b) CoA-Thioester Hydrolase (CHY1; Synonym: *β*-Hydroxyisobutyryl-CoA Hydrolase)**

In our analysis, one UR tag of this class showing FC of 2.8 (FC_T_ = FC_D_; Table 3) was identified. This peroxissomal metabolic enzyme is needed for valine catabolism and fatty acid b-oxidation. Analyzing freezing sensitive *Arabidopsis* mutants (*chy1-10*) after cold acclimation, Dong et al. [51] observed that the disruption of CHY1 function leads to an excess of methylacrylyl-CoA, causing accumulation of *Reactive Oxygen Species* (ROS), electrolyte leakage, impairing cold-induced gene expression. Additionally, methylacrylyl-CoA may be sequestered in the peroxisome leading to localized changes in this sub cellular region and influencing peroxisome-derived signals after cold-induction. Potential alterations in auxin response or homeostasis in the *chy1* mutant may contribute to the impaired cold stress tolerance of the mutant, since peroxisome-defective mutants showed resistance to the inhibitory effects of exogenous IBA, analogous to the IAA molecule (a hormone that inhibits the root elongation and promotes lateral root formation).

**(c) Glutathione Transferase (GST; EC 2.5.1.18)**

GSTs encode an ancient, heterogeneous, and widely distributed protein group in living organisms catalyzing a variety of reactions [52], including hormonal metabolism, vacuolar sequestration of anthocyanin, tyrosine metabolism, hydroxyperoxide detoxification, and regulation of apoptosis [52, 53]. In our study, one UR tag (SD92627) associated to GST showed a significant expression modulation (FC_T_ = 5.7 and FC_D_ = 2.4; Table 3). GST expression is induced by a wide variety of stresses, as oxidative stress [54], xenobiotic-type of stresses [55], and dehydration [56]. Expression of *TaGSTU1B (Triticum aestivum) *was induced by drought stress in four genotypes investigated, but high transcript amounts were detected only in drought-tolerant genotypes [57]. George et al. [58] reported the subcellular localization and the ability of GST from *Prosopis juliflora *(*PjGSTU1*), a drought-tolerant woody Fabaceae species, to confer drought tolerance in transgenic tobacco. Ji et al. [59] working with tobacco plants overexpressing a GST gene from *Glycine soja* showed six-fold higher GST activity enhanced dehydration tolerance than wild-type plants.

**(d) Serine Hydroxymethyltransferase (SHMT; EC 2.1.2.1)**

The SHMT genic family comprises five genes in *A. thaliana *[60] bearing both cytosolic and mitochondrial isoforms in eukaryotes [61] with activity associated to the Serine and Glycine metabolism (EMBL, 2010). In our evaluation two SHMT candidates [SD243418 (FC_T_ = 4.0; FC_D_ = ns); SD179937 (FC_T_ = 3.8; FC_D_ = 2.5); Table 3] were identified. According to Moreno et al. [62], *Arabidopsis* SHMT1 functions in the photorespiratory pathway and influences resistance to biotic and abiotic stresses. The *Arabidopsis* SHMT1 mutant (*shmt1-1*) showed enhanced susceptibility to pathogens, as well as to abiotic stresses (50 mM NaCl and high light intensity). The reduced activity in *shmt1-1* mutant appears to hinder the ability of the plant to cope with any kind of additional stress, compromising the cellular mechanisms during oxidative stress. In proteome analysis [63], ten out of twelve drought responsive proteins identified from rice leaf sheaths were upregulated including an SHMT. The authors suggested that SHMT was induced for protection from oxidative degradation under drought stress.

**(e) Peptidyl-Prolyl Cis-Trans Isomerase (PPIase)**

It is also known as rotamases or immunophilins (cyclophilins included), which is an enzyme superfamily with catalytic function, facilitating metabolism regulation through a chaperone or a cis-trans isomerization of proline residues during protein folding [64, 65]. A UR tag (SD169158) showing an FC_T_ = 3.2 (Table 3) concerns a potential PPIase. In plants PPIases have been associated with the response to adverse environmental conditions. Using contrasting genotypes of *Sorghum bicolor* under water deficit, Sharma and Singh [64] observed a significant increase in leaf- and root-PPIase activity in the drought-tolerant cultivar. Similarly, various rice PPIases were differentially expressed under water deficit and salinity (200 mM NaCl) stresses [65]. Also, a correlation with plant hormones was pointed out by Godoy et al. [66] working with cyclophilins (CyPs) of *Solanum tuberosum*. CyPs are ubiquitous proteins with an intrinsic enzymatic activity of PPIase that catalyzes the rotation of X-Pro peptide bonds. *StCyP* mRNA accumulation was stimulated by the application of abscisic acid (ABA) and methyl jasmonate (MeJA) in potato tubers. The accumulation of *StCyP* transcripts was also detected when the potato tubers were exposed to heat-shock treatment.

**(f) Viviparous14**

It is a key enzyme involved in the biosynthesis of the phytohormone abscisic acid [67], represented in our analysis by the SD140270 tag with FC_T_ of 3.2 (Table 3). Viviparous genes are encoded in the process of plant vivipary, also reported as early germination. Of the 15 genes described so far for maize, 12 control specific steps in ABA biosynthesis [68, 69], with *vip14* (viviparous-14), associated to the control of final steps of ABA synthesis, encoding a 9-cis-epoxycarotenoid dioxygenase 1 (NCED1) enzyme that catalyzes the cleavage of the C40 neoxanthin chain into the C15 ABA skeleton xanthoxin [70]. Maize mutants for the *nced1* gene have strongly reduced kernel ABA content [71] while in *Arabidopsis*, NCED1 overexpression conferred a significant increase in ABA accumulation in the plant and also in drought tolerance [72]. Wan and Li [73] demonstrated that the expression of *AhNCED1* gene in peanut plants was significantly upregulated by dehydration and high salinity (250 mmol·L^−1^ NaCl).

**(g) Branched-Chain Amino Acid Transaminase**

BCATs are enzymes that play a crucial role in the metabolic pathway of BCAAs (branched-chain amino acids that include leucine, isoleucine, and valine) by catalyzing the last step of synthesis and the initial step of degradation of these amino acids [74]. Plants contain a small family of *bcat* genes, which have been characterized in *Solanum tuberosum* (potato), *Hordeum vulgaris,* and *A. thaliana *[75, 76]. Malatrasi et al. [77] evaluated the role of these enzymes in the drought tolerance process. In this study, the transcriptional levels of *Hvbcat-1*, in *H. vulgaris*, increased seven folds (results obtained by double checking with RTqPCR) after progressive drought stress (up to 14 days of water deprivation). Physiologically, the authors associated the overregulation to the activation of the BCAAs catabolism, since this is the first enzyme in the branched-chain amino acid (BCAA) catabolic pathway. In high concentrations, these amino acids are toxic to the cells; therefore, activation of their catabolism may play an important role as detoxification mechanism. In our analysis, two UR tags annotated as BACTs were identified exhibiting an expressive modulation of the FC, mainly for the SD237939 tag (FC of 6.8 for both FC_T_ and FC_D_; Table 3), while the other tag (SD238059) showed an FC of 2.4 (FC_T_ = FC_D_).

**(h) Allene Oxide Synthase**

AOS is the first enzyme in the pathway leading to the biosynthesis of Jasmonic acid (JA), catalyzing the production of unstable allene epoxides that cyclize to form cyclopentenone acids, the precursors for JA [78]. Three tags of this category were identified (SD270381, SD272257, SD63148) being upregulated in most comparisons (Table 3). For example, the SD270381 tag presented high FC values in both T and D comparisons. The overexpression of AOSs has been observed also in other drought assays, as reported by Ozturk et al. [79] and Talamè et al. [80] with barley (*H. vulgare*) and peanut (*Arachis hypogaea*) [81].

**(i) Na+/H+ Antiporter**

Membrane proteins involved in the Na+ and H+ transport of both eukaryotes and prokaryotes act in the homeostasis maintenance of such ions [82]. In our analysis, the SD213044 tag, annotated as potential Na+/H+ antiporter, was overexpressed in both analyzed contrasting situations (Table 3). Assays evaluating those proteins under salinity stress showed that these salt-responsive genes may be able to activate the expression of drought-related genes in the tolerance acquisition [83]. Thus, ions are stored in vacuoles, acting as osmolytes, decreasing the hydric potential of the cell. Evaluations with transgenic plants overexpressing those genes, including* Petunia hybrida *[83], *A. thaliana *[84], and *A. hypogaea* [85], conferred higher tolerance to dehydration under drought and salinity.

**(j) Glutathione Peroxidase (EC 1.11.1.9)**

In the present approach, a UR GPX candidate (SD219102) was overexpressed FC_T_ of 3.2 (Table 3). These enzymes are known as cell protectors against oxidative damage generated by reactive oxygen species [86]. They present a very broad distribution in the cell, occurring in several subcellular compartments [87]. Miao et al. [88] suggested that ATGPX3 might play dual and distinctive roles in H_2_O_2_ homeostasis, acting as a general scavenger and relaying the H_2_O_2_ signal, and also as an oxidative ABA signal transducer during drought stress signaling. Their differential regulation during biotic and abiotic stresses was reported by Navrot et al. [87], indicating their importance for plant breeding.

**(l) Serine-Threonine Kinase SAPK1 (Also Known as JNK)**

It belongs to the MAPK family [89], including important proteins active in the osmosensory signal transduction pathways in cells exposed to osmotic stress [90]. A wheat candidate (*W55a*) with about 90% homology to rice SAPK1 was evaluated by Xu et al. [91]. Transgenic *Arabidopsis* plants overexpressing W55a exhibited higher tolerance to drought, being also upregulated by salt, exogenous abscisic acid, salicylic acid, ethylene, and methyl jasmonate. In addition, W55a transcripts were abundant in leaves, but not in roots or stems, under environmental stresses. Expression of *SAPK* members analyzed by RNA gel blot hybridization with samples of leaves (blades and sheath), roots, and treatments with ABA (50 *μ*M), NaCl (150 mM), or mannitol (600 mM) showed that *SAPK1* was upregulated by all three treatments in both roots and leaves, although the effect of ABA was weaker than those of the other two treatments. *SAPK6* was weakly upregulated by all treatments in the blades and the sheaths, and weakly by ABA or NaCl but strongly by mannitol treatment in the roots [92]. Overexpressed candidates analyzed here (SD129463; FC_T_ = 3.0; Table 3) included an SAPK1 as well as a second tag matching SAPK6 (SD87319; FC_T_ = 2.4 and FC_D_ = 1.6; Table 3), both in roots, indicating their activation also in this tissue.

**(m) Delta-1-Pyrroline-5-Carboxylate Synthetase**

P5CS is an enzyme that catalyzes the initiation of the proline biosynthesis in plants [93]. The excessive production of this amino acid would increase the osmotolerance in plants [94]. Rice plants transformed with the P5CS gene underwent 10 days of irrigation withdrawal with higher growth rates, when compared to the control group [94]. Effects of salt in transgenic tobacco transformed with *P5CS* gene revealed the overexpression of P5CS after 24–48 h exposure to NaCl (300 mM), when compared with non-transgenic plants under the same stress [95]. Transgenic lines of petunia [96] and tobacco [97] with enhanced accumulation of proline showed also high drought tolerance. Transcripts involved in amino acid metabolism, such as *P5CS*, *OAT* and *AS*, were also induced more than 10 folds during the identification of drought-responsive genes during sucrose accumulation and water deficit in sugarcane [98]. In our study, a UR tag (SD251703) showing an expressive induction (FC_T_ 7.1) was annotated as a potential *P5CS* candidate (Table 3).

## 4. Concluding Remarks

The present report is the first to analyze contrasting sugarcane accessions under drought stress with a combination of the high-throughput transcriptome profiling SuperSAGE technology coupled with a next-generation sequencing platform. This approach allowed the identification of many potential target candidates in the drought stress response. The adopted methodology of annotation and GO categorization revealed the success of the work in accessing genes from very different pathways, ranging from those controlling the perception and first reaction against the stress (as transcription factors) to those known as classic genes of the osmotic stress (as *P5CS*). The number of induced tags (213) with GO categorization and high modulation is surprising, especially considering the short time (24 h) after drought stress application. Besides, a high number of important gene candidates with no hits (145)—probably completely new to the research community—will demand additional efforts for the recognition of their function. Validation procedures as well as transient expression assays are planned for future works, aiming to collaborate with breeding and biotechnological approaches for the benefit of the sugarcane culture, especially facing the scenario of future climate changes.

## Figures and Tables

**Table 1:** Total number of differentially expressed (DE; *P* ≤ 0.05) up- or downregulated tags observed in different contrasting SuperSAGE root libraries from sugarcane under drought stress (24 hours without irrigation) as compared with negative control (irrigated materials).

Contrasting	Upregulated	Downregulated	DE	Total
T (TD × TC)	12,179	12,482	24,661	152,049
S (SD × SC)	12,085	16,339	28,424	141,946
D (TD × SD)	15,591	12,269	27,860	148,657
C (TC × SC)	12,961	16,342	29,303	148,631

TD: bulk of tolerant genotypes under stress; TC: bulk of tolerant genotypes without stress (control); SD: bulk of sensitive genotypes under stress; SC: bulk of sensitive genotypes without stress (control).

**Table 2:** Total number of sugarcane upregulated (UR) root tags observed on contrasting SuperSAGE libraries when associated with ESTs classified by Gene Ontology (GO) in the subcategories related to abiotic stress response.

Response against	GO categories	Exclusive UR tags	Common UR tags after comparison	
		T	T and D	T and S
Water deprivation	0009414	20	25	11
Heat and cold	0009408; 0009409	19	54	18
Osmotic stress	0006970	32	57	19
Oxidative stress	0006979	19	27	6
Abscisic acid stimulus	0009737	24	37	12
Jasmonic acid stimulus	0009753	5	19	6

EST: expressed sequence tag; contrast of libraries [T (TD *versus* TC); D (TD *versus* SD); S (SD *versus* SC)]; Libraries [TD: drought-tolerant bulk under stress; TC: tolerant bulk control; SD: drought-sensitive bulk under stress; SC: sensitive bulk control].

**Table 3:** Upregulated SuperSAGE tags associated via Gene Ontology (GO) to abiotic stress, with fold change for T (FC_T_ = TD/TC) and D (FC_D_ = TD/SD) comparisons for tag frequencies in the sugarcane roots libraries, as well as the annotation of the best aligned EST.

Tag	GO	FC_T_	FC_D_	Annotation
SD159390	AJ	5.6	ns	50S ribosomal prot. L5, chloroplast
SD191288	AJ	2.9	2.3	AP2 domain transcription factor EREBP
SD122727	AJ	2.2	2.7	Bet v I allergen-like
SD75453	AJ	2.8	2.8	Chromatin-remodeling factor CHD3
SD123546	AJ	1.6	ns	Chromatin-remodeling factor CHD3
SD9608	AJ	2.4	3.3	Initiator-binding prot.; ibp
SD15756	AJ	1.9	1.6	OSK1; SNF1-related prot. Kinase
SD108270	AJ	6.4	2.6	ZIM motif family prot.
SD258836	AJ	3.1	ns	ZIM motif family prot.
SD108269	AJ	2.9	2.1	ZIM motif family prot.
SD133809	AJ	2.8	2.6	ZIM motif family prot.
SD196399	AJ/Os	3.2	3.2	P18; Nucleoside diphosphate kinase I; NDK1
SD169158	AJ/Os/Ox	3.2	ns	Peptidyl-prolyl cis-trans isomerase
SD252082	AJ/WH	3.6	3.6	Auxin-induced prot.
SD237930	AJ/WH/Os	2.4	ns	18S ribosomal RNA gene
SD282917	AJ/WH/Os	1.5	1.6	ABA responsive element binding factor 2
SD237939	AJ/WH/Os	6.8	6.8	Branched-chain-amino-acid aminotransf.
SD238059	AJ/WH/Os	2.4	2.4	Branched-chain-amino-acid aminotransf.
SD140270	AJ/WH/Os	3.2	ns	viviparous-14
SD237936	AJ/WH/Os/Ox	2.8	2.8	Ribosomal prot. L28e domain cont. prot.
SD178862	AJ/WH/Os/Ox	2.8	2.8	18S ribosomal RNA gene
SD203616	WH	1.3	ns	RAP2-like prot.
SD246714	WH	2.8	2.8	CoA-thioester hydrolase CHY1
SD286424	WH	1.4	2.6	ERF/AP2 domain cont. transcription factor
SD279457	WH	2.8	ns	Mitochondrial uncoupling prot. 2
SD107875	WH	3.7	5.2	Nucleic acid binding
SD191687	WH	2.1	6.9	RAPB prot.
SD147607	WH	5.6	ns	Salt tolerance prot.
SD109060	WH	1.4	1.3	Transposable element Mu1 sequence
SD199146	Os	1.1	1.1	Alpha tubulin-4^a^
SD54073	Os	2.3	6.8	Calreticulin-like prot.
SD102228	Os	6.8	6.8	Endo-1,4-beta-glucanase Cel1
SD80163	Os	4.7	ns	Endo-1,4-beta-glucanase Cel1
SD13344	Os	1.9	ns	Eukaryotic translation if 2 alpha sub family
SD182876	Os	4.4	ns	Phosphopantetheine adenylyl transf. dephospho CoA kinase
SD129463	Os	3.0	ns	Serine/threonine-prot. kinase SAPK1
SD87319	Os	2.4	1.6	Serine/threonine-prot. kinase SAPK6
SD270381	Ox	6.5	4.5	Allene oxide synthase
SD272257	Ox	2.4	ns	Allene oxide synthase
SD63148	Ox	2.0	2.5	Allene oxide synthase
SD113907	Ox	2.4	2.4	Brassinosteroid biosynthesis-like prot.
SD219102	Ox	3.2	ns	Glutathione peroxidase
SD213044	Ox	2.2	2.4	Na+/H+ antiporter
SD54454	Ox	2.0	3.1	Nicotianamine aminotransferase A
SD125582	Ox	3.8	1.9	Nicotinate phosphoribosyltransferase-like
SD122742	Ox	3.2	ns	Nucleotide repair prot.
SD102844	Ox	1.6	ns	Peroxidase precursor
SD17103	Ox	6.4	ns	Tyrosine/nicotianamine aminotransf. family
SD17107	Ox	2.5	ns	Tyrosine/nicotianamine aminotransf. family
SD17108	Ox	1.8	ns	Tyrosine/nicotianamine aminotransf. family
SD151691	WH/Os	1.8	1.6	DRF-like transcription factor DRFL2a
SD9805	WH/Os	25.0	25.0	Glycine-rich RNA binding prot.
SD9802	WH/Os	14.7	14.7	Glycine-rich RNA binding prot.
SD9806	WH/Os	13.1	13.1	Glycine-rich RNA binding prot.
SD9767	WH/Os	2.8	2.8	Glycine-rich RNA binding prot.
SD9803	WH/Os	2.4	2.4	Glycine-rich RNA binding prot.
SD9800	WH/Os	2.4	2.4	Glycine-rich RNA binding prot.
SD9801	WH/Os	1.1	ns	Glycine-rich RNA binding prot.
SD108120	WH/Os	6.0	6.0	Glycine-rich RNA-binding prot.
SD108115	WH/Os	1.3	1.3	Glycine-rich RNA-binding prot. 2; GRP2
SD264077	WH/Os	3.6	ns	Membrane integral prot.
SD92627	WH/Ox	5.7	2.4	Glutathione transferase III
SD243418	WH/Ox	4.0	ns	Serine hydroxymethyltransferase
SD179937	WH/Ox	3.8	2.5	Serine hydroxymethyltransferase
SD21923	WH/Ox	1.3	1.7	Whitefly-induced gp91-phox
SD184083	Os/Ox	3.2	3.2	Delta-1-pyrroline-5-carboxylate dehydrog.
SD8088	Os/Ox	3.2	ns	MutT domain prot.-like
SD251703	Os/Ox	7.1	ns	P5cs; delta 1-pyrroline-5-carboxylate synth.

Libraries [TD: drought-tolerant bulk under stress; TC: tolerant bulk control; SD: drought-sensitive bulk under stress]; ns: fold change of tag not significant (*P* ≤ 0.05). WH: response to water deprivation and to heat/cold; Os: response to osmotic stress, Ox: response to oxidative stress; AJ: response to abscisic acid stimulus and to jasmonic acid stimulus.
